# Decellularization Strategies for Regenerative Medicine: From Processing Techniques to Applications

**DOI:** 10.1155/2017/9831534

**Published:** 2017-04-30

**Authors:** Anna Gilpin, Yong Yang

**Affiliations:** Department of Chemical and Biomedical Engineering, West Virginia University, Morgantown, WV 26506, USA

## Abstract

As the gap between donors and patients in need of an organ transplant continues to widen, research in regenerative medicine seeks to provide alternative strategies for treatment. One of the most promising techniques for tissue and organ regeneration is decellularization, in which the extracellular matrix (ECM) is isolated from its native cells and genetic material in order to produce a natural scaffold. The ECM, which ideally retains its inherent structural, biochemical, and biomechanical cues, can then be recellularized to produce a functional tissue or organ. While decellularization can be accomplished using chemical and enzymatic, physical, or combinative methods, each strategy has both benefits and drawbacks. The focus of this review is to compare the advantages and disadvantages of these methods in terms of their ability to retain desired ECM characteristics for particular tissues and organs. Additionally, a few applications of constructs engineered using decellularized cell sheets, tissues, and whole organs are discussed.

## 1. Introduction

Tissue and organ failure is currently one of the biggest health issues our society faces. Arising from disease or trauma, complete treatment typically requires the reparation or replacement of the affected organ. Conventional practices utilize organs from either live or deceased donors for the procedure of transplantation. However, the deficit between donors and patients requiring a new organ has grown substantially in recent years, so much that there are more than 100,000 patients on waiting lists for organs in the United States alone [1]. This epidemic has led researchers to explore alternative methods of treatment, in particular tissue engineering, which seeks to reduce organ specificity and thus alleviate the necessity for organ donation.

Tissue engineering aims at replacing or regenerating human tissues or organs in order to restore or establish normal function [2]. The tissue engineering triad consists of three main factors, the cells, signaling molecules, and scaffold, which support and rely upon one another. Specifically, the scaffold, together with integrated signaling molecules, provides structural, biochemical, and biomechanical cues to guide and regulate cell behavior and tissue development. Scaffolds can be prepared through a variety of methods and materials, both synthetic and natural. Synthetic scaffolds are beneficial in that their structure and mechanical properties can be manipulated and controlled with the goal of producing an optimal environment for a particular cell type or cell set. Among these properties, matrix stiffness and topography show profound influences on cell growth and differentiation. Stiffer polymers like poly(lactic-co-glycolic acid) (PLGA) have been used in cartilage and bone tissue engineering [3, 4], while softer hydrogels, benefitting from their tunable mechanical properties and adjustable composition, have been used for soft tissues [5]. Interestingly, differentiation towards a desirable lineage can be facilitated on a matrix having stiffness similar to that of the natural tissue [6, 7]. The nanotopography of the scaffold also plays an important role in cell regulation. In addition to inducing pronounced changes to cell morphology and gene expression [8–11], nanotopography can regulate the differentiation of stem cells into certain lineages such as neuron [8, 12, 13], muscle [14], and bone [15, 16]. Thus, cell behavior can be regulated through tailoring the scaffold's properties. Technologies like electrospinning and 3D printing enable the control of these properties through optimization in the materials and processing parameters. Electrospun constructs, which consist of arranged polymeric nanofibers, have been utilized in engineering several tissue structures, including vascular grafts [17], bone [18], and myocardium [19]. Furthermore, 3D printing in scaffold design allows for the integration of features like vasculature [20, 21] and the control of scaffold architecture [22], using synthetic polymers or bioinks. These methods show great promise in their ability to control key properties of scaffolds and to subsequently manipulate cell behavior for tissue engineering.

Because many challenges are associated with preparing synthetic scaffolds that recapitulate the complexity of the cell microenvironment, there has been increasing interest in utilizing naturally derived extracellular matrix (ECM) itself. This biologic scaffold is obtained through the process of decellularization. The ultimate goal of decellularization is to rid the ECM of native cells and genetic materials such as DNA while maintaining its structural, biochemical, and biomechanical cues. The decellularized ECM can then be repopulated with a patient's own cells to produce a personalized tissue. Decellularized ECM has been successfully used to recreate various types of tissues and organs including blood vessel [23–25], heart valve [26, 27], cornea [28–30], trachea [31], esophagus [32], urinary bladder [33], kidney [34, 35], liver [36–39], lung [40, 41], and heart [42, 43].

**Figure 1:**
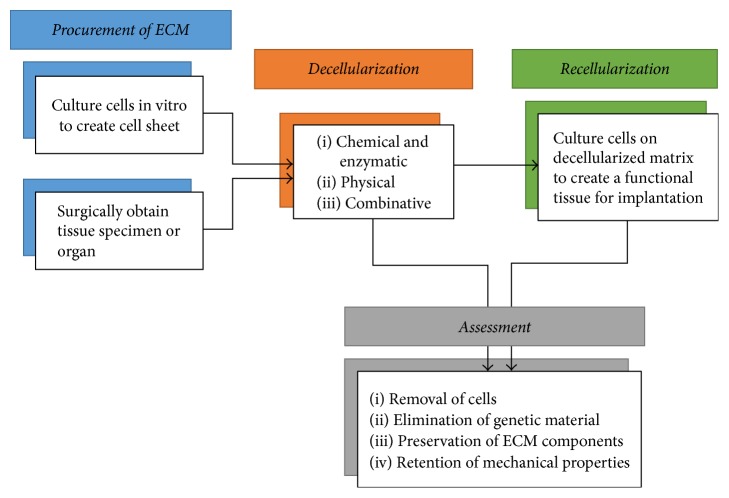
Process of engineering tissues using decellularized ECM.

Figure 1 outlines the overall process of developing engineered tissues or organs from decellularization to recellularization. In the decellularization process, chemical and enzymatic, physical, or combinative methods are utilized to remove cells and DNA from the tissue while preserving its structural and regulatory proteins. To assess the quality of decellularized ECM, four aspects of the ECM are measured: removal of cells, elimination of genetic material, preservation of protein content, and retention of mechanical properties. Cell and genetic material removal is critical in preventing immunorejection of the construct to seeded cells. The criteria for assessing the efficacy of removal of these components is suggested as follows: the decellularized ECM must have (1) less than 50 ng double-stranded DNA (dsDNA) per mg ECM dry weight, (2) less than 200 bp DNA fragment length, and (3) no visible nuclear material by 4′,6-diamidino-2-phenylindole (DAPI) staining [44]. Additionally, protein content remaining in the ECM should be evaluated, with emphasis on the structural proteins like collagen, fibronectin, and laminin; glycosaminoglycans (GAGs); and growth factors. Moreover, the mechanical properties including elastic modulus and tensile strength, depending on the application, should match the original tissue. In this review, decellularization strategies are evaluated based upon their efficacy in these four areas, in addition to the toxicity and overall efficiency of the technique, followed by a few applications of decellularized ECM.

## 2. Decellularization Strategies

Decellularization has been performed through chemical, physical, or combinative methods. An evaluation of these strategies will first focus on the removal of cells and genetic material followed by the maintenance of structural proteins. In addition, mechanical properties and ultrastructure of the tissue or organ before and after treatment will be compared. Emphasis will be placed upon the method's ability to retain the characteristics necessary for successful recreation of the tissue or organ.  Table 1 summarizes the chemical and enzymatic agents and mechanical techniques used for decellularization.

### 2.1. Chemical and Enzymatic Approaches

Several types of chemicals have been used in decellularization, including surfactants, acids, and bases. Surfactants, the most common decellularizing agents, typically work by lysing cells through disarranging the phospholipid cell membrane [57]. These agents are classified based upon their charge, as they are ionic, nonionic, or zwitterionic. Acids like peracetic acid and bases like sodium hydroxide solubilize the cell membrane and nuclear material by utilizing their intrinsically charged properties.

#### 2.1.1. Surfactants

The widely used ionic surfactant, sodium dodecyl sulfate (SDS), has been successful in a number of applications in decellularization because of its ability to efficiently remove cells and genetic material. For example, it was the major agent used in the whole rat heart perfusion decellularization [42]. SDS treatments have met the standard requirements of complete cell removal and elimination of at least 90% of host DNA in several types of tissues and organs, including rat forearm [58], porcine cornea [30], porcine myocardium [59], porcine heart valve [47], porcine small intestine [45], porcine kidney [34], human vein [60], rat, porcine, and human lungs [40, 46], and human heart [61]. Administering SDS to whole organs via perfusion allowed for the maintenance of ultrastructure. Specifically, continuously perfusing the solution through the vasculature not only preserved the structure of the blood vessels but also helped to prevent the deformation of alveoli [40]. This aspect was vital for the nourishment of seeded stem cells [62] and ultimately for the recovery of the organ's original function.

Although SDS can successfully remove unwanted native constituents of the tissue, it can be damaging to the structural and signaling proteins. For instance, the collagen in SDS-treated heart valves became compacted [47], and the decellularized ECM of human and porcine lungs appeared more fibrous than the structure of the smooth native tissue [46]. The fibrous structure of human hearts decellularized using SDS; however, it was not affected, as the anisotropic properties resembled those of native myocardium [61]. GAGs and growth factors like vascular endothelial growth factor (VEGF) were also diminished by SDS treatment, which can affect the biochemical cues regulating cell function. Such a decrease in GAGs, however, was likely a result of the location of these molecules in the cell membrane, which was destroyed in the decellularization process [39]. Damaging structural proteins and components not only can prevent the cells from inhabiting the tissue as before but also prevents the full retention of its mechanical properties. This result is particularly prevalent in thinner tissues and cell sheets. After being treated with a 0.5 wt% SDS solution, fibroblast cell sheets showed an 80% decrease in elastic modulus [55]. As the SDS concentration increased, the elastic and viscous moduli decreased, indicating the damaging nature of SDS. In the case of decellularized porcine myocardium, the tissue became stiffer, as the tensile strength was twenty times greater than the native tissue [59]. However, because collagen type IV was maintained, the tissue exhibited extensibility, a property critical for its desired function as a cardiac patch. SDS is also cytotoxic; therefore, it is imperative that the tissue is thoroughly washed to ensure the viability of reseeded cells [45]. While most surfactant-treated tissues must typically be washed with solutions like phosphate buffered saline (PBS), SDS is more difficult to remove due to its ionic nature. The required extensive wash process following treatment is another disadvantage of SDS as a decellularizing agent.

The nonionic surfactant Triton X-100 is oftentimes utilized to remove the remnant SDS. This practice has been especially prevalent in the perfusion decellularization of whole organs [39, 40, 42, 45, 58, 61]. Not only is Triton X-100 beneficial in the wash process, but also it is commonly used as a decellularizing agent alone. As it is nonionic, it is less harsh than SDS and ultimately less damaging to the structural integrity of the tissue. In conjunction with ammonium hydroxide, it completely removed all DNA and maintained a greater amount of collagen I compared to SDS treatments [34]. Additionally, the tissue's ultrastructure and mechanical properties were well preserved following the decellularization process [48].

Sodium deoxycholate (SD) is another ionic surfactant that works by solubilizing the cell membrane. Unlike SDS, SD produced scaffolds that were highly biocompatible, as cells seeded on the SD-decellularized matrices exhibited higher metabolic activity compared to those decellularized via SDS [45]. Complete cell removal by using SD was observed in a perfusion treatment of rat lungs [40], as well as an agitation treatment of porcine heart valve leaflets [47]. Although SD is nondamaging, it can cause the agglutination of DNA on the tissue's surface [49]. To address this issue, a 4% SD solution was combined with the enzyme, deoxyribonuclease I (DNase I), to break down the tissue's native DNA. This particular protocol was used for a variety of structures including blood vessels [49, 63], tracheas [50, 64], diaphragm [65], aortic root [66], and small intestines [45, 51]. However, despite the fact that the function of DNase I is to break down DNA fragments, a significant amount of DNA remained in the ECM following treatment [45, 49–51, 66]. As a result, more extensive wash processes must be performed in order to eliminate DNA present in the tissue to prevent immunorejection. Additional treatment cycles have also been shown to eliminate a greater percentage of DNA, as was the case in the decellularization of the diaphragm, which exhibited a 95% decrease in native genetic material after three cycles [65]. In addition, structural proteins such as fibronectin and laminin were retained, and the position of collagen fibers was preserved in the treatment of porcine and rat tracheas [50, 64], as well as the rat diaphragm [65]. This property is important for the trachea, in particular, since its shape and structure affect the resistivity of the airway. In addition to ECM proteins, von Willebrand factor was found to be present in the decellularized aortic root, a protein key to maintaining hemostasis, and the mechanical properties remained similar. However, lower peak pressures were observed, signifying an alteration in valve closure functioning. Furthermore, the compliance of the tissue was maintained in the SD/DNase I treatment of blood vessels, which is crucial for their ability to contract and dilate [63]. In the case of decellularized lungs, a greater amount of myosin was retained when using SD compared to that with other surfactant treatments [40], and collagen and elastin were well preserved in the heart valve leaflets [47]. The muscle fiber arrangement and collagen fiber alignment in the diaphragm remained following three SD/DNase cycles, which enabled similar mechanical function to native tissue [65]. These results highlight the ability of SD to retain the structural proteins necessary for the tissue's function.

The zwitterionic, nondenaturing detergent, 3-[(3-cholamidopropyl)dimethylammonio]-1-propanesulfonate (CHAPS), has been applied in both immersion and perfusion decellularization procedures. Human- and porcine-derived lung tissues [40, 46] and rat lungs [40, 41] were treated with 8 mM CHAPS solutions, each exhibiting complete decellularization by the histological analysis. However, cytoplasmic proteins remained, indicating that not all cellular debris was removed [40]. ECM proteins like collagen and elastin were preserved, thereby allowing the lung tissue to retain its compliance. This aspect was likely due to the nondenaturing properties of CHAPS, which are highly beneficial in lung applications due to the requirement that the tissue expand and contract with ease. The ultrastructure of the lung, airways, and vasculature, as well as the structure of the alveoli, was retained in whole lung experiments [40, 41]. While CHAPS excelled in the areas of protein and structural maintenance, other detergents like SDS tended to demonstrate greater efficiency in reducing the level of residual DNA. Thus, further wash steps to eliminate the immunogenic potential of the tissue treated with CHAPS may improve the overall efficacy of this detergent.

#### 2.1.2. Acids and Bases

Acid- and base-containing protocols utilize agents like peracetic acid and techniques like reversible alkaline swelling. Peracetic acid is a highly corrosive and strong oxidizer oftentimes used for sterilization. Thinner tissues such as the small intestine submucosa (SIS) [45] and urinary bladder [52] have been treated with peracetic acid as a decellularizing agent. While it was shown that the SIS was biocompatible following treatment, the cells were not entirely removed [45]. In addition, the mechanical properties of the tissue were altered with a significant increase in yield stress and elastic modulus, particularly in the longitudinal rather than circumferential direction. A similar effect was observed in the treatment of urinary bladder matrix and submucosa, as a stiffer ECM was produced in the longitudinal direction resulting from differences in collagen fiber alignment [52]. These results imply an alteration in function of the tissue following peracetic acid treatment, which indicates that its use may not be suitable for tissues in which expandability and compliance are desired properties. In another study regarding the decellularization of bovine pericardium, tridecyl alcohol ethoxylate, a nonionic surfactant, was used by itself and with a calcium oxide alkaline solution [48]. Used alone, the tissue was completely void of cells and the viscoelasticity and ultrastructure remained the same. With the addition of the alkaline solution, swelling resulted from the induced negative charge on collagen in the tissue, which was subsequently reversed with ammonium sulfate. While this protocol similarly eliminated cellular and genetic material and maintained the ultrastructure of the tissue, the swelling caused a reduction in the tissue's GAG content and viscoelasticity. Thus, it was ultimately harsher than the tridecyl alcohol ethoxylate alone and provided no significant benefits in terms of reducing immunogenicity.

#### 2.1.3. Enzyme-Assisted Decellularization

Like the use of DNase I to prevent the agglutination of DNA in SD treatments, other types of enzymes have been used in decellularization protocols to supplement the chemicals' properties. For example, Triton X-100 and SD were used in combination with DNase in the decellularization of both normal and emphysematous human lungs [67] and porcine heart valves [68] in order to break down remnant DNA fragments so as to limit potential immunogenicity in vivo. A perfusion and immersion approach was utilized in the lung treatment [67]. Collagens type I and IV, fibronectin, and laminin were maintained; however, the GAG content was significantly lower following treatment. While the functional smooth muscle proteins, myosin and actin, were preserved, elastin decreased. The overall ultrastructure of the lung, including the alveolar septum, was maintained. In the decellularization of the heart valves, a three-day wash cycle was performed, and the decellularized ECM subsequently underwent enzymatic digestion with both DNase and RNase [68]. Cellular debris was fully eliminated and collagen and elastin were maintained. Another detergent-enzymatic treatment involving the use of an SDS/SD solution and endonuclease for equine carotid artery decellularization highlighted potential complications that may arise with xenogeneic scaffolds [69]. While no visible nuclei were observed and DNA was significantly reduced following treatment, several antibody-triggering proteins remained. The presence of these distinguishing factors indicated failure to reduce the scaffold's immunogenicity and, therefore, to produce a universally compatible matrix. In addition, the amount of glyceraldehyde 3-phosphate dehydrogenase (GAPDH), a glycolytic enzyme, and smooth muscle actin decreased with the SDS/SD endonuclease treatment. Another multistep protocol utilized trypsin/ethylenediaminetetraacetic acid (EDTA), SDS, Triton X-100, peracetic acid/ethanol, and DNase to decellularize porcine rectus abdominis muscle [70]. Cells and DNA were sufficiently removed and laminin, collagen type IV, and fibronectin remained following treatment. Additionally, the decellularized muscle displayed mechanical strength similar to the native muscle. The ultrastructure, vasculature, and neural channels were intact as well.

Trypsin is an enzyme commonly used with EDTA which works by breaking the cell-matrix adhesions. Used in the treatment of porcine pulmonary valves, complete cell and genetic material removal was observed after 24 hours [53]. At shorter treatment times, particularly at eight hours, the cell removal was incomplete. A longer treatment time, however, led to decreased GAG, collagen, and elastin content and, subsequently, mechanical strength. In addition, due to the properties of trypsin/EDTA, the salt- and acid-soluble collagens were poorly preserved. Because of the inefficiency of trypsin/EDTA-only treatment, several of the aforementioned detergents have been combined in certain protocols. In another study of porcine heart valve decellularization, the tissues were treated with Triton X-100 and DNase and RNase following trypsin/EDTA exposure [47]. Cell nuclei did not remain and elastin fibers were preserved intact. Collagen fibers, on the other hand, deformed, a result which can be detrimental to the heart valve's structural function. In porcine trachea decellularization, trypsin was supplemented to the SD/DNase I in a five-cycle protocol [71]. Each cycle involved the addition of 1% trypsin (3 hr at 4°C), 4% SD (4 hr at room temperature), and DNase I (3 hr at room temperature) with wash cycles in between each new solution. No cellular debris remained and collagen and elastic properties were maintained. The most significant result of this decellularization treatment was the elimination of chondrocytes, which was likely due to the trypsin breaking down the chondronectin fibers in the trachea's cartilage. Many other treatments involving this structure have been unsuccessful in this regard [50, 64]. Therefore, chemical approaches can be improved by combining them with enzymatic treatments that may remove the unwanted cellular and genetic components of the ECM. However, their efficacy is ultimately dependent upon the maintenance of ECM features that are critical in regenerating the desired function of the particular tissue.

### 2.2. Mechanical Approaches

While chemical and enzymatic approaches are the most widely applied decellularization methods, there are concerns regarding the possible toxicity of the chemicals and destruction of ECM proteins. Other methods that can physically or mechanically decellularize the ECMs are therefore being developed. These methods include temperature and pressure treatments that work to eliminate cells through a combination of lysing the cells and destroying cell-matrix adhesive proteins. In particular, physical treatments include the use of freeze-thaw, high hydrostatic pressure, or supercritical carbon dioxide (CO_2_) to fully remove a tissue's constituent cells and genetic materials. While each specific method is unique, every mechanical decellularization protocol involves a wash process to remove any cellular debris that may remain. In many cases, the washing step is critical in determining the efficacy of the procedure.

#### 2.2.1. Freeze-Thaw

Freezing and thawing tissues lyses, and subsequently eliminates, the cells to produce a decellularized matrix. Freeze-thaw procedures oftentimes involve alternating between freezing temperatures around −80°C and biological temperatures around 37°C. Specific protocols can be altered by increasing the temperature difference or changing the number of freeze-thaw cycles performed. Two particular freeze-thaw studies involved the decellularization of fibroblast cell sheets carried out with three cycles [55] and canine lumbar spinal segments carried out with one cycle [56]. In both instances of freeze-thaw, the collagen and GAG content, as well as the mechanical strength, were similar to those of the native specimen [55, 56]. However, 88% of the DNA in the fibroblast cell sheets remained following treatment [55]. These results indicate that the ECM scaffold resulting from this procedure may produce an immunogenic response in vivo. Therefore, although freeze-thaw procedures are beneficial in their retention of biochemical components and biomechanical properties, they can potentially lead to immunorejection due to the insufficient removal of genetic materials.

#### 2.2.2. High Hydrostatic Pressure

High hydrostatic pressure (HHP) has become an increasingly prevalent method that applies pressures greater than 600 MPa to destroy cell membranes. In one study, porcine corneas were decellularized using HHP at 980 MPa for 10 minutes at 10 or 30°C [28]. The same procedure was also carried out on porcine blood vessels [23]. In both tissues, the high-pressure treatment destroyed the cells, yet it left behind DNA remnants. Because HHP alone failed in this regard, the wash solutions utilized in both studies contained DNase I to break down fragments in order to prevent immunorejection [23, 28]. The cornea wash solution also contained the glucose polymer, dextran, in order to reduce swelling caused by the submersion in the solution [28]. Glycerol was added in order to help maintain its transparency and elastic moduli, two properties crucial to the cornea's function in the eye.

Difference in the temperature can lead to alterations in protein content and structure. At 10°C, both collagen and GAG content were better maintained in the cornea decellularization than at 30°C [28]. However, ice formation occurred at 10°C at HHP, which led to the destruction of tissue structure [23]. Furthermore, the high pressure itself was shown to denature ECM proteins, as observed in the deformation of collagen and elastin fibers in the decellularized blood vessels and consequently the decrease in ultimate tensile strength by approximately 50% [23]. Therefore, although HHP treatment is beneficial in its short treatment time and ability to sterilize the tissue through its destruction of bacterial and viral membranes, it requires an extensive wash process and can alter the structural and mechanical properties of the tissue.

#### 2.2.3. Supercritical Carbon Dioxide

With the unique transport properties, that is, a liquid-like density and a gas-like diffusivity, supercritical fluids have been used in extraction applications in industry [72–74]. In particular, supercritical CO_2_ has a critical temperature of 31.1°C and a critical pressure of 7.40 MPa, which is biologically permissive. Hence, supercritical CO_2_ has been used in the decellularization of aortic tissue, where the tissue was treated at 15 MPa and 37°C [54]. Moreover, due to the diffusivity of CO_2_, the solvent can be quickly released and does not remain within the tissue, preventing the need for extensive wash procedures usually required for the processes involving surfactants. Because CO_2_ is nonpolar, ethanol was added as an entrainer to remove the polar phospholipid cell membranes. This addition proved effective, since neither the cell nucleus nor membrane remained in the tissue after treatment. Collagen and elastin content, and subsequently mechanical strength, were not altered with treatment, demonstrating the nondeforming capabilities of supercritical CO_2_.

### 2.3. Combined Methods

Because each aforementioned technique has its advantages and disadvantages, several techniques have been combined to complement one another with the goal of retaining desired characteristics in the engineered tissue. For example, mechanical methods are typically less damaging to the tissue's structure; however, they fail to meet the requirements for immunogenicity. On the other hand, surfactants at low concentrations or enzymes used alone may not completely remove all cellular debris. To this regard, combining the two treatments in a multistep process can yield a decellularized ECM appropriate for its particular application. While the combined method may demand more chemicals and a longer processing time than single-treatment protocols, each constituent parameter needs to be optimized to suit a particular tissue. These tissue-specific protocols can thus be more practical and effective.

Thicker tissues, in particular those composed of multiple layers, oftentimes require multistep decellularization procedures involving chemical, enzymatic, and mechanical treatments. Adipose, or fat tissue, is one type of thick tissue and is desired in reconstructive surgeries. While its characteristics are simple compared to more complex tissues such as lung or heart, it is necessary to maintain adipose tissue's mechanical properties and biochemical factors in order to regulate adipogenesis. In one particular method, a multistep process was carried out on porcine adipose tissue [75]. The procedure began by mechanically massaging the tissue while it was frozen to facilitate cell removal. The tissue then underwent an enzymatic treatment of trypsin/EDTA and surfactant treatments of Triton X-100 and SD. It was sterilized using ethanol and peracetic acid, and polar remnants were solubilized using n-propanol. Following the 16-step treatment, complete cell, DNA, and lipid removal was observed. Additionally, both collagen type IV and laminin fibers were maintained, as was the majority of the GAG content. While this process damaged collagen I fibers and reduced the levels of vascular endothelial growth factor (VEGF) and transforming growth factor-beta (TGF-*β*), the tissue did facilitate adipogenesis. Another treatment of adipose tissue involved a five-day process of multiple freeze-thaw cycles and alternating between two different enzymatic digestion solutions (one containing trypsin and the other containing DNase II, RNase III, and lipase IV), in addition to polar solvent extractions [76]. Cellular debris was completely removed throughout the tissue, and laminin and collagen I fibers were maintained. In addition, part of the vasculature remained, which would aid in the recellularization and vascularization of the reconstructed tissue.

Multistep chemical, enzymatic, and mechanical combinative methods have been proven to be detrimental to different properties of some tissues. In a protocol involving the treatment of porcine cartilage disks, two different hypotonic buffers were used, the first supplemented with 100 mM KCl, 5 mM MgCl_2_, and 100 mM dithiothreitol (DTT) and the second supplemented with 0.5% SDS, in order to solubilize the cell membranes [77]. The tissue was then subjected to hyaluronidase to increase its porosity, as well as alternating cycles of freeze-thaw and a nuclease digestion. To further increase porosity, the tissue also underwent sonication in sodium hydroxide. Creating a more porous scaffold was desired in order to aid in DNA removal and subsequent recellularization. The method was effective in removing a majority of cells and DNA, as well as maintaining collagen and ultrastructure of the tissue. However, several mechanical properties decreased as a result of increased porosity. In the treatment of whole rat lungs, two different mechanical methods were utilized in combination with surfactants and enzymes [78]. Four freeze-thaw cycles were performed; then the lungs were placed in a continuously circulating bioreactor with 1% SDS. After five weeks, the lungs were removed and treated with a nuclease digestion. Characterization of the resultant ECM revealed the absence of cellular debris and the maintenance of a majority of the collagen and elastin content. Collagen type I fibers, however, no longer had the same fibrous pattern, and collagen type IV, laminin, and fibronectin were significantly reduced. Such a difference in ECM composition was likely due to the large amount of time the lungs underwent treatment.

## 3. Assessment of Decellularization

### 3.1. Immunogenicity

Reducing the scaffold's immunogenicity is one of the most critical requirements of decellularization. This particular aspect has been crucial in preventing the use of decellularized ECM as scaffolds in clinical applications. Ideally, xenogeneic scaffolds may be used, as they are highly abundant and thus have the potential to be manufactured. If their immunogenicity is not sufficiently reduced, they may be rejected in vivo, leading to functional failure and the need for immediate replacement or removal. The two components capable of inducing an immunogenic response include remnant genetic materials such as DNA and RNA and antigens. In terms of eliminating genetic materials, Crapo and colleagues have suggested that the decellularized ECM should contain less than 50 ng dsDNA per mg ECM and DNA fragment lengths less than 200 bp [44]. Detergents including SDS and Triton X-100 have met the first criterion, as they remove greater than 90% of remnant DNA [30, 34, 40, 45–47, 58, 60]. Other detergents such as SD and CHAPS, however, are less successful in this regard [40, 41, 45, 49–51]. For the second criterion, endonucleases including DNase and RNase have been used to break down nucleic acid fragments. While these enzymes successfully reduce the length of fragments and subsequently prevent significant immunogenic responses, they do little to separate the fragments from the ECM. Furthermore, mechanical approaches including the use of freeze-thaw and HHP left behind DNA remnants, suggesting the need for more extensive wash procedures [23, 28, 55].

Native antigens must also be reduced in the scaffolds to prevent immunorejection. Hyperacute rejection of scaffolds, occurring shortly after implantation and caused by circulating antibodies within the host, and acute immune rejection, occurring days to weeks after implantation, are of particular concern [79]. This aspect is oftentimes not considered in decellularization; however, it is critical to analyze the presence of triggering entities prior to implantation in vivo. Specific components that may be measured include alpha-gal epitopes which have the potential to activate the complement cascade immune response and major histocompatibility (MHC) complexes present on the cell membrane, which can lead to T cell and natural killer cell responses [79]. While these are immunogenic molecules that provide primarily adverse effects to the host, ECM structural proteins like collagen VI have indicated potential immunogenicity [80]. Thus, while these proteins are desired to maintain the structure and mechanical properties of the engineered construct, they may be overtly specific to their native organism. Additional strategies to limit their immunogenic effect, in particular recellularizing the ECM with autologous cells, are therefore necessary to prevent host rejection.

### 3.2. Mechanical Properties

In regenerating a tissue or organ via decellularization, maintaining the mechanical characteristics of the native tissue is of vital importance in ensuring proper functionality. Primary properties of interest include elastic modulus, viscous modulus, tensile strength, and yield strength; however, the most crucial properties ultimately depend on the nature of the tissue or organ's desired function. In particular, stiffness is desired in the design of a trachea to provide an unobstructed airway. Furthermore, the anisotropic or isotropic characteristics of the tissue are also necessary to modulate, as they can oftentimes dictate the orientation of reseeded cells: such is the case with cardiomyocytes in myocardium regeneration [81]. These properties are primarily governed by the ECM structural proteins collagen, laminin, fibronectin, and elastin. Each decellularization strategy produces different effects on these proteins; therefore, the technique used must be chosen based upon the tissue biomechanics necessary for proper function.

Both chemical and mechanical methods have been shown to damage the structural proteins of the ECM. In particular, SDS has been shown to alter the ECM's microstructure by causing collagen to become compacted [47] and producing a more fibrous matrix [46]. More pronounced changes in the structure and mechanical properties tend to be correlated with increased concentrations of the detergent. This effect was observed in the decrease in elastic modulus of fibroblast cell sheets treated with SDS [55]. Other detergents like Triton X-100, SD, and CHAPS, however, sufficiently maintained key ECM proteins and mechanical properties. Additionally, acids and bases have been shown to alter the compliance of tissues, as peracetic acid increased the elastic modulus of the SIS and bladder [45, 52] and reversible alkaline swelling decreased the viscoelasticity of bovine pericardium [48]. The amount of time the tissue is exposed to the decellularizing agent also plays an important role in damaging the structural proteins. In particular, porcine pulmonary valves treated with EDTA and trypsin exhibited a decrease in mechanical strength at longer treatment times, which were necessary for complete cell removal [53]. This effect is likely a result of changes in collagen content and fiber orientation, as EDTA has been shown to diminish salt- and acid-soluble collagens [47, 53]. Mechanical treatments better maintain these properties; however, the parameters at which the treatments are carried out must be optimized accordingly. Freeze-thaw treatments sufficiently preserve the mechanical strength of the original tissue [55, 56]; however, this may be impacted by further cycles. High hydrostatic pressure has a risk of denaturing ECM proteins [23]. In addition, when performed at lower temperatures, ice may form within the tissue, thus leading to greater deformation. Therefore, exposure to each of these treatments must be performed under the mildest conditions necessary to fully decellularize and remove immunogenic constituents of the ECM.

## 4. Recellularization

Recellularization of the decellularized ECM must also be optimized in order to produce a functional tissue or organ. The cell type used to repopulate the matrix and method of recellularization is largely dependent on the complexity of the cell sheet, tissue, or organ of interest. Cell sheets for skin grafts or blood vessels may only require a single cell type, whereas whole organs such as the heart or liver necessitate the seeding of multiple cell types. Furthermore, recellularization of cell sheets can be accomplished by simply applying the cell suspension onto the monolayer surface, and three-dimensional constructs can be created through alternating between the cell suspension and additional cell sheets as in the “sandwich model” for cartilage construction [82, 83]. Multilayer tissues such as the colon may be separated in order to expose individual layers for precise injection [84]. In addition, sonication has been utilized to facilitate cell seeding through inducing pore formation within the tissue [77, 85]. For thicker tissues and whole organs, however, the cells may be injected directly into the tissue or perfused through the vasculature of the construct. The latter method is especially beneficial following perfusion decellularization in which detergent solutions are pumped directly into the vasculature for a given period of time [38, 39, 42]. Additionally, reendothelializing the vasculature of decellularized constructs has also been shown to improve the organization of seeded cells. For example, endothelial cells were reintroduced via the aorta only, the brachiocephalic artery (BA) only, or both the aorta and BA of a decellularized rat heart prior to the introduction of cardiomyocytes, which increased their contractibility [43]. The combined approach proved to be more effective in endothelializing the vasculature and subsequently limited its thrombogenicity. The bioreactor environment has also been shown to influence the success of recellularization, as a rotating wall vessel bioreactor facilitated greater cell proliferation and viability of two different cell types seeded within decellularized mouse lungs over static conditions [86]. Furthermore, acellular scaffolds may be introduced into the body so that autologous cells may naturally repopulate the ECM.

## 5. Applications

Decellularization has been performed on a variety of cell sheets, tissues, or organs, depending on the necessary replacement or regenerative treatment.  Table 2 highlights decellularization strategies most commonly employed for various ECM types. Because of their differing structure and function, the strategy for decellularization and recellularization of each must be accommodated for their level of complexity. Here, we discuss how different decellularization methods have been applied to regenerate various cell sheets, tissues, and whole organs.

### 5.1. Cell Sheets

Cell sheets can be prepared and subsequently decellularized to be used in a number of applications. They typically consist of a single cell type that can be used alone to facilitate regeneration of minor injuries or combined with many sheets and structured to form complex constructs. For example, to generate an ECM to promote periodontium regeneration, periodontal ligament cell sheets were prepared and then decellularized to conserve the native growth factor-, collagen-, and fibronectin-laden matrix [87]. Moreover, many cell sheets derived from cartilage sections have been combined in a “sandwich model” to generate two-dimensional cartilage constructs. In one study, 10 *μ*m decellularized cartilage slices were alternated with chondrocytes, with the cell suspension sandwiched between the decellularized matrices, for a total of 20 layers [82]. In another similar model, bone-marrow mesenchymal stem cells were seeded instead so they could differentiate into chondrocytes [83]. In both cases, cartilage constructs capable of being implanted in vivo were formed.

Another promising application involving the creation of blood vessels from fibroblast cell sheets emphasizes the ability of simple decellularized matrices to form more complex structures. In one study, fibroblast monolayer cell sheets were prepared and rolled into vessels before being decellularized and seeded with endothelial cells [24]. Specifically, cell sheets were cultured for three weeks in growth media supplemented with ascorbic acid and then rolled around mandrels of diameters comparable to natural blood vessels. These tubular structures were then cultured for an additional four weeks and decellularized via hypoosmotic shock in deionized water over one week. Next, they were placed in a rotating bioreactor and injected with endothelial cells for four hours. Constructs were conditioned in the bioreactor for an additional week before undergoing mechanical testing. While the engineered vessel's mechanical properties were comparable to natural blood vessels, these properties, which include burst pressure and suture retention, decreased by 24% over three months. These results indicate potential disadvantages for long-term implantation. Another study utilizing fibroblast cell sheets for blood vessel formation detailed decellularizing the matrices and reseeding them with smooth muscle cells prior to rolling them into cylinders [88]. Because smooth muscle cells were used, histamine-induced contraction was observed. Thus, each of these methods exhibits the potential of functional, responsive blood vessel creation using decellularized cell sheets.

### 5.2. Tissues

Decellularization methods have been used on different types of tissues either to examine the treatment on a less complex tissue or to create a scaffold for simpler applications. For example, adipose ECM has been utilized in testing breast cancer treatments by providing simulated in vivo microenvironments [89]. Additionally, adipose tissue has been frequently decellularized and then reseeded with a patient's own adipose-derived stem cells in order to create constructs used in reconstructive surgeries for structural or cosmetic purposes [75, 76, 90]. Bone reconstruction has also been tested using bone-derived ECM to facilitate osteogenesis or the differentiation of marrow stromal cells into osteoblasts [91]. Advancements in this area have the potential to reduce the need for bone grafts. Similarly, decellularization of cartilage tissue has been performed, as it is desired for treatments relating to joint degeneration, namely, arthritis [56, 92]. Each of these applications highlights the benefits of tissue decellularization to create in vitro environments for drug testing and supplementing in vivo tissue regeneration.

In another example of adipose tissue regeneration, adipose tissue was successfully decellularized, recellularized, and implanted in vivo. Human adipose tissue was obtained from reconstructive surgeries and decellularized using a protocol that first required three freeze-thaw cycles, a two-day agitation period, and two four-hour saline solution washes of different concentrations repeated over two days. The sample was then treated with 0.25% trypsin/EDTA for two hours, washed, and submersed in isopropyl alcohol overnight. Finally, the sample underwent treatment in 1% Triton X-100 for three days and was washed in ultrapure water for two days and PBS for one day [90]. Decellularized ECM was seeded with human adipose-derived stem cells, and the resultant constructs were implanted into the backs of nude rats. Samples were then tested periodically over eight weeks. Decellularized adipose tissue that had not yet been recellularized was also implanted and incubated for 30 days. Results showed that the constructs did not elicit an immunogenic response, and they promoted vascularization and subsequent regeneration of adipose tissue.

### 5.3. Whole Organs

The complexity of organ function requires the most extensive decellularization and reconstruction techniques, in particular necessitating the maintenance of the organ's ultrastructure and vasculature. A variety of organs have been engineered in this way and have subsequently been tested in vivo on the small animal level. In one example, the vasculature of decellularized porcine bladder matrices was resurfaced using epithelial progenitor cells. These scaffolds supported reseeded bladder smooth muscle and urothelial cells, and the resultant bladders were successfully implanted for one to three hours in pigs [33]. The vasculature itself is also commonly utilized as a means of injecting desired cells to repopulate the decellularized scaffold, as seen with the heart [42] and liver [38, 39]. Basic functionality of each organ was also observed, as contraction was seen in the heart [42], and albumin and urea production was seen in the liver [38, 39]. However, long-term studies have been lacking in the area of bioscaffold-produced engineered whole organs, so the ability of the organ to perform its function over time is necessary to evaluate.

One particular study observed an engineered pancreas in mice that had been achieved through perfusion decellularization [93]. The isolated mouse pancreases were injected with 0.5% SDS until they became translucent followed by 1% Triton X-100 and a solution of Benzonase before being washed. Scaffolds were then recellularized with two types of pancreatic cells for a culture period of five days before being implanted for 14 days. The first type of cells, MIN-6 cells, was introduced via the hepatic portal vein and seeded through the vasculature in three steps. The second cell type, AR42J cells, was seeded through the pancreatic duct. These cells successfully repopulated the pancreas, and insulin gene expression was observed to be upregulated, indicating appropriate function. Additionally, implantation in mice revealed that the construct was biocompatible and did not elicit a significant immunogenic response.

Clinical applications of decellularized ECM are becoming more prevalent; however, they have been limited to less complex tissues primarily functioning in structural or reconstructive roles. Several FDA-approved products on the market aimed at tissue regeneration and replacement are derived from xenogeneic or allogeneic decellularized ECM, including LifeCell's AlloDerm® Regenerative Tissue Matrix (human dermal graft), DSM's Meso BioMatrix® Surgical Mesh (porcine mesothelium), and CryoLife's SynerGraft® (human pulmonary heart valve). The former two scaffolds serve to facilitate cell proliferation, whereas the lattermost product serves as a replacement for the existing structure. Furthermore, clinical trials have been carried out for more complex structures. For example, the first tissue-engineered trachea to be implanted was generated using decellularization [31]. Although the patient's native trachea experienced stenosis near the interface of the engineered construct within five years following transplantation, the vascularized and recellularized construct remained intact and did not provoke a significant immunogenic response [94]. While long-term clinical studies using decellularized ECM as scaffolds are minimal, these results show their promise in the development of more accessible treatments for a variety of applications.

## 6. Outlook

While great advancements are being made in regenerative medicine through decellularization, it is essential that protocols be further developed to optimize the process. Current decellularization methods are beneficial in some regards but oftentimes lack in others. Ideal methods will produce cell- and genetic material-free ECM that retains important structural, biochemical, and biomechanical properties crucial to its inherent function. Several methods may be optimized in such a way as to achieve this balance, specifically in eliminating the components individualized to the tissue which could cause an immunogenic response, while maintaining those that will support and regulate the reconstruction of a new tissue. In particular, developing methods that consist of combined strategies with chemicals, enzymes, and mechanical techniques may improve decellularization efficiency and limit the negative effects caused by simpler methods. This improvement may be achieved through dynamic processes in which parameters are adjusted over time based upon the component in need of removal or maintenance. Thus, the tissue may be examined and assessed at predetermined time points throughout treatment. For example, supercritical CO_2_ may be utilized for cell lysis; then the ECM may undergo brief exposure to lower concentrations of surfactants to eliminate remnant cellular materials. Further enzymatic or wash agents can then be applied. The goal is ultimately to administer the minimum amount of harsh chemicals or mechanical treatments so as to prevent unnecessary damage to the ECM's microstructure and ultrastructure.

By optimizing the decellularization process, tissue and organ scaffolds derived from humans and animals may be utilized, thus minimizing the donor-patient specificity required to ensure compatible transplants. However, truly bridging the gap between donors and patients in need of transplants through bioscaffold tissue engineering will require the development of scaled-up methods, since current methods are usually limited to small tissues or organs. In addition, recellularization methods will need to be improved in order to distribute the proper cell types uniformly through the tissue and enable sufficient nutrient and oxygen delivery for optimal cell viability. Finally, with the continuous innovation in decellularization strategies and advancements in uses for human induced pluripotent stem cells, off-the-shelf tissues and organs may be made available, thereby aiding in, and potentially eliminating, the ever-increasing need for transplants.

## Figures and Tables

**Table 1:** Summary of decellularization agents and techniques.

Category	Agents and techniques	Mechanism/description	Significant effects	References
Chemical and enzymatic	(1)* Surfactants*	*Lyse cells by disarranging the phospholipid membrane*		
	Sodium dodecyl sulfate (SDS)	(i) Ionic	(i) Cytotoxic: requires extensive wash process	[45]
			(ii) Alters microstructure (i.e., collagen fibers)	[46, 47]
	Triton X-100	(i) Nonionic	(i) Less damaging to structure of tissue than ionic surfactants	[34, 48]
		(ii) Commonly used with ammonium hydroxide		
	Sodium deoxycholate (SD)	(i) Ionic	(i) Causes agglutination of DNA when used without DNase	[49]
		(ii) Commonly used with DNase	(ii) Remnant DNA fragments	[45, 49–51]
	CHAPS	(i) Zwitterionic	(i) Maintains structural ECM proteins	[40]
			(ii) Remnant cytoplasmic proteins	[40]
			(iii) Maintains ultrastructure	[40, 41]
	(2)* Acids and bases*	*Solubilize cell membrane by utilizing charged properties*		
	Peracetic acid	(i) Highly corrosive	(i) Insufficient cell removal	[45]
		(ii) Oftentimes used for sterilization	(ii) Increases stiffness of ECM	[45, 52]
	Ethylenediaminetetraacetic acid (EDTA)	(i) Commonly used with trypsin	(i) Decreases salt- and acid-soluble ECM proteins	[53]
	Reversible alkaline swelling	(i) Induces negative charge on collagen to cause swelling	(i) Alters mechanical properties	[48]
		(ii) Used with tridecyl alcohol ethoxylate		
	(3)* Enzymes*	*Typically used to supplement other chemical & mechanical treatments*		
	Trypsin	(i) Breaks cell-matrix adhesions		
		(ii) Commonly used with EDTA		
	Deoxyribonuclease (DNase)	(i) Breaks down DNA fragments		
		(ii) Commonly used with SD		
	Ribonuclease (RNase)	(i) Breaks down RNA fragments		

Mechanical	High hydrostatic pressure (HHP)	(i) Pressures greater than 600 MPa applied to lyse cells	(i) Remnant DNA fragments	[23, 28]
			(ii) Denatures ECM proteins	[23]
	Supercritical carbon dioxide	(i) Applies CO_2_ at pressures above 7.40 MPa and temperatures above 31.1°C	(i) Requires entrainer to remove polar phospholipid membrane	[54]
			(ii) Maintains ECM proteins & mechanical properties	[54]
	Freeze-thaw	(i) Alternate between freezing temperatures (−80°C) and biological temperatures (~37°C)	(i) Maintains ECM proteins & mechanical properties	[55, 56]
			(ii) Remnant DNA	[55]

**Table 2:** Summary of ECM type treated using main decellularization strategies.

Agents and techniques	Cell sheet/tissue/organ
SDS	Fibroblast cell sheet [55], rat forearm [58], porcine cornea [30], porcine myocardium [59], porcine heart valve [47], porcine small intestine (SIS) [45], porcine kidney [34], human vein [60], rat, porcine, and human lungs [40, 46], human heart [61]
Triton X-100	Bovine pericardium [48], porcine kidney [34]
SD/DNase	Porcine blood vessel [63], bovine blood vessel [49], rat trachea [64], porcine trachea [50], rat small intestine [51], porcine SIS [45]
CHAPS	Rat lung [40, 41], human and porcine lung [40, 46]
Peracetic acid	Porcine SIS [45], porcine urinary bladder [52]
EDTA/trypsin	Porcine pulmonary valve [47, 53]
Freeze-thaw	Fibroblast cell sheet [55], canine lumbar spinal segment [56]
High hydrostatic pressure	Porcine cornea [28], porcine blood vessel [23]

## References

[1] Health Resources and Services Administration (2016). *Organ Procurement and Transplantation Network*.

[2] Mason C., Dunnill P. (2008). A brief definition of regenerative medicine. *Regenerative Medicine*.

[3] Uematsu K., Hattori K., Ishimoto Y. (2005). Cartilage regeneration using mesenchymal stem cells and a three-dimensional poly-lactic-glycolic acid (PLGA) scaffold. *Biomaterials*.

[4] Wu Y.-C., Shaw S.-Y., Lin H.-R., Lee T.-M., Yang C.-Y. (2006). Bone tissue engineering evaluation based on rat calvaria stromal cells cultured on modified PLGA scaffolds. *Biomaterials*.

[5] Drury J. L., Mooney D. J. (2003). Hydrogels for tissue engineering: scaffold design variables and applications. *Biomaterials*.

[6] Engler A. J., Griffin M. A., Sen S., Bönnemann C. G., Sweeney H. L., Discher D. E. (2004). Myotubes differentiate optimally on substrates with tissue-like stiffness: pathological implications for soft or stiff microenvironments. *Journal of Cell Biology*.

[7] Saha K., Keung A. J., Irwin E. F. (2008). Substrate modulus directs neural stem cell behavior. *Biophysical Journal*.

[8] Yim E. K. F., Pang S. W., Leong K. W. (2007). Synthetic nanostructures inducing differentiation of human mesenchymal stem cells into neuronal lineage. *Experimental Cell Research*.

[9] Gerecht S., Bettinger C. J., Zhang Z., Borenstein J. T., Vunjak-Novakovic G., Langer R. (2007). The effect of actin disrupting agents on contact guidance of human embryonic stem cells. *Biomaterials*.

[10] Downing T. L., Soto J., Morez C. (2013). Biophysical regulation of epigenetic state and cell reprogramming. *Nature Materials*.

[11] Jeon H., Koo S., Reese W. M., Loskill P., Grigoropoulos C. P., Healy K. E. (2015). Directing cell migration and organization via nanocrater-patterned cell-repellent interfaces. *Nature Materials*.

[12] Lee M. R., Kwon K. W., Jung H. (2010). Direct differentiation of human embryonic stem cells into selective neurons on nanoscale ridge/groove pattern arrays. *Biomaterials*.

[13] Moe A. A. K., Suryana M., Marcy G. (2012). Microarray with micro- and nano-topographies enables identification of the optimal topography for directing the differentiation of primary murine neural progenitor cells. *Small*.

[14] Dang J. M., Leong K. W. (2007). Myogenic induction of aligned mesenchymal stem cell sheets by culture on thermally responsive electrospun nanofibers. *Advanced Materials*.

[15] Dalby M. J., Gadegaard N., Tare R. (2007). The control of human mesenchymal cell differentiation using nanoscale symmetry and disorder. *Nature Materials*.

[16] Oh S., Brammer K. S., Li Y. S. J. (2009). Stem cell fate dictated solely by altered nanotube dimension. *Proceedings of the National Academy of Sciences of the United States of America*.

[17] Hasan A., Memic A., Annabi N. (2014). Electrospun scaffolds for tissue engineering of vascular grafts. *Acta Biomaterialia*.

[18] Jang J.-H., Castano O., Kim H.-W. (2009). Electrospun materials as potential platforms for bone tissue engineering. *Advanced Drug Delivery Reviews*.

[19] Kitsara M., Agbulut O., Kontziampasis D., Chen Y., Menasché P. (2017). Fibers for hearts: a critical review on electrospinning for cardiac tissue engineering. *Acta Biomaterialia*.

[20] Paulsen S. J., Miller J. S. (2015). Tissue vascularization through 3D printing: will technology bring us flow?. *Developmental Dynamics*.

[21] Lee V. K., Kim D. Y., Ngo H. (2014). Creating perfused functional vascular channels using 3D bio-printing technology. *Biomaterials*.

[22] An J., Teoh J. E., Suntornnond R., Chua C. K. (2015). Design and 3D printing of scaffolds and tissues. *Engineering*.

[23] Funamoto S., Nam K., Kimura T. (2010). The use of high-hydrostatic pressure treatment to decellularize blood vessels. *Biomaterials*.

[24] Tondreau M. Y., Laterreur V., Gauvin R. (2015). Mechanical properties of endothelialized fibroblast-derived vascular scaffolds stimulated in a bioreactor. *Acta Biomaterialia*.

[25] Shimizu K., Ito A., Arinobe M. (2007). Effective cell-seeding technique using magnetite nanoparticles and magnetic force onto decellularized blood vessels for vascular tissue engineering. *Journal of Bioscience and Bioengineering*.

[26] Syedain Z., Reimer J., Schmidt J. (2015). 6-month aortic valve implantation of an off-the-shelf tissue-engineered valve in sheep. *Biomaterials*.

[27] Reimer J. M., Syedain Z. H., Haynie B. H. T., Tranquillo R. T. (2015). Pediatric tubular pulmonary heart valve from decellularized engineered tissue tubes. *Biomaterials*.

[28] Hashimoto Y., Funamoto S., Sasaki S. (2010). Preparation and characterization of decellularized cornea using high-hydrostatic pressurization for corneal tissue engineering. *Biomaterials*.

[29] Oh J. Y., Kim M. K., Lee H. J., Ko J. H., Wee W. R., Lee J. H. (2009). Processing porcine cornea for biomedical applications. *Tissue Engineering—Part C: Methods*.

[30] Pang K., Du L., Wu X. (2010). A rabbit anterior cornea replacement derived from acellular porcine cornea matrix, epithelial cells and keratocytes. *Biomaterials*.

[31] Macchiarini P., Jungebluth P., Go T. (2008). Clinical transplantation of a tissue-engineered airway. *The Lancet*.

[32] Totonelli G., Maghsoudlou P., Georgiades F. (2013). Detergent enzymatic treatment for the development of a natural acellular matrix for oesophageal regeneration. *Pediatric Surgery International*.

[33] Schultheiss D., Gabouev A. I., Cebotari S. (2005). Biological vascularized matrix for bladder tissue engineering: matrix preparation, reseeding technique and short-term implantation in a porcine model. *Journal of Urology*.

[34] Sullivan D. C., Mirmalek-Sani S.-H., Deegan D. B. (2012). Decellularization methods of porcine kidneys for whole organ engineering using a high-throughput system. *Biomaterials*.

[35] Song J. J., Guyette J. P., Gilpin S. E., Gonzalez G., Vacanti J. P., Ott H. C. (2013). Regeneration and experimental orthotopic transplantation of a bioengineered kidney. *Nature Medicine*.

[36] Mazza G., Rombouts K., Rennie Hall A. (2015). Decellularized human liver as a natural 3D-scaffold for liver bioengineering and transplantation. *Scientific Reports*.

[37] Soto-Gutierrez A., Zhang L., Medberry C. (2011). A whole-organ regenerative medicine approach for liver replacement. *Tissue Engineering Part C: Methods*.

[38] Baptista P. M., Siddiqui M. M., Lozier G., Rodriguez S. R., Atala A., Soker S. (2011). The use of whole organ decellularization for the generation of a vascularized liver organoid. *Hepatology*.

[39] Uygun B. E., Soto-Gutierrez A., Yagi H. (2010). Organ reengineering through development of a transplantable recellularized liver graft using decellularized liver matrix. *Nature Medicine*.

[40] Gilpin S. E., Guyette J. P., Gonzalez G. (2014). Perfusion decellularization of human and porcine lungs: bringing the matrix to clinical scale. *Journal of Heart and Lung Transplantation*.

[41] Petersen T. H., Calle E. A., Colehour M. B., Niklason L. E. (2012). Matrix composition and mechanics of decellularized lung scaffolds. *Cells Tissues Organs*.

[42] Ott H. C., Matthiesen T. S., Goh S.-K. (2008). Perfusion-decellularized matrix: using nature's platform to engineer a bioartificial heart. *Nature Medicine*.

[43] Robertson M. J., Dries-Devlin J. L., Kren S. M., Burchfield J. S., Taylor D. A. (2014). Optimizing recellularization of whole decellularized heart extracellular matrix. *PLoS ONE*.

[44] Crapo P. M., Gilbert T. W., Badylak S. F. (2011). An overview of tissue and whole organ decellularization processes. *Biomaterials*.

[45] Syed O., Walters N. J., Day R. M., Kim H.-W., Knowles J. C. (2014). Evaluation of decellularization protocols for production of tubular small intestine submucosa scaffolds for use in oesophageal tissue engineering. *Acta Biomaterialia*.

[46] O'Neill J. D., Anfang R., Anandappa A. (2013). Decellularization of human and porcine lung tissues for pulmonary tissue engineering. *Annals of Thoracic Surgery*.

[47] Zhou J., Fritze O., Schleicher M. (2010). Impact of heart valve decellularization on 3-D ultrastructure, immunogenicity and thrombogenicity. *Biomaterials*.

[48] Mendoza-Novelo B., Avila E. E., Cauich-Rodríguez J. V. (2011). Decellularization of pericardial tissue and its impact on tensile viscoelasticity and glycosaminoglycan content. *Acta Biomaterialia*.

[49] Meezan E., Hjelle J. T., Brendel K., Carlson E. C. (1975). A simple, versatile, nondisruptive method for the isolation of morphologically and chemically pure basement membranes from several tissues. *Life Sciences*.

[50] Partington L., Mordan N. J., Mason C. (2013). Biochemical changes caused by decellularization may compromise mechanical integrity of tracheal scaffolds. *Acta Biomaterialia*.

[51] Maghsoudlou P., Totonelli G., Loukogeorgakis S. P., Eaton S., De Coppi P. (2013). A decellularization methodology for the production of a natural acellular intestinal matrix. *Journal of Visualized Experiments*.

[52] Gilbert T. W., Wognum S., Joyce E. M., Freytes D. O., Sacks M. S., Badylak S. F. (2008). Collagen fiber alignment and biaxial mechanical behavior of porcine urinary bladder derived extracellular matrix. *Biomaterials*.

[53] Schenke-Layland K., Vasilevski O., Opitz F. (2003). Impact of decellularization of xenogeneic tissue on extracellular matrix integrity for tissue engineering of heart valves. *Journal of Structural Biology*.

[54] Sawada K., Terada D., Yamaoka T., Kitamura S., Fujisato T. (2008). Cell removal with supercritical carbon dioxide for acellular artificial tissue. *Journal of Chemical Technology and Biotechnology*.

[55] Xing Q., Yates K., Tahtinen M., Shearier E., Qian Z., Zhao F. (2015). Decellularization of fibroblast cell sheets for natural extracellular matrix scaffold preparation. *Tissue Engineering—Part C: Methods*.

[56] Elder B. D., Kim D. H., Athanasiou K. A. (2010). Developing an articular cartilage decellularization process toward facet joint cartilage replacement. *Neurosurgery*.

[57] Nazari M., Kurdi M., Heerklotz H. (2012). Classifying surfactants with respect to their effect on lipid membrane order. *Biophysical Journal*.

[58] Jank B. J., Xiong L., Moser P. T. (2015). Engineered composite tissue as a bioartificial limb graft. *Biomaterials*.

[59] Wang B., Borazjani A., Tahai M. (2010). Fabrication of cardiac patch with decellularized porcine myocardial scaffold and bone marrow mononuclear cells. *Journal of Biomedical Materials Research—Part A*.

[60] Schaner P. J., Martin N. D., Tulenko T. N. (2004). Decellularized vein as a potential scaffold for vascular tissue engineering. *Journal of Vascular Surgery*.

[61] Guyette J. P., Charest J. M., Mills R. W. (2016). Bioengineering human myocardium on native extracellular matrix. *Circulation Research*.

[62] Orlando G., Baptista P., Birchall M. (2011). Regenerative medicine as applied to solid organ transplantation: current status and future challenges. *Transplant International*.

[63] Pellegata A. F., Asnaghi M. A., Stefani I. (2013). Detergent-enzymatic decellularization of swine blood vessels: insight on mechanical properties for vascular tissue engineering. *BioMed Research International*.

[64] Baiguera S., Del Gaudio C., Kuevda E., Gonfiotti A., Bianco A., Macchiarini P. (2014). Dynamic decellularization and cross-linking of rat tracheal matrix. *Biomaterials*.

[65] Piccoli M., Urbani L., Alvarez-Fallas M. E. (2016). Improvement of diaphragmatic performance through orthotopic application of decellularized extracellular matrix patch. *Biomaterials*.

[66] Friedrich L. H., Jungebluth P., Sjöqvist S. (2014). Preservation of aortic root architecture and properties using a detergent-enzymatic perfusion protocol. *Biomaterials*.

[67] Wagner D. E., Bonenfant N. R., Parsons C. S. (2014). Comparative decellularization and recellularization of normal versus emphysematous human lungs. *Biomaterials*.

[68] Rieder E., Kasimir M.-T., Silberhumer G. (2004). Decellularization protocols of porcine heart valves differ importantly in efficiency of cell removal and susceptibility of the matrix to recellularization with human vascular cells. *The Journal of Thoracic and Cardiovascular Surgery*.

[69] Böer U., Lohrenz A., Klingenberg M., Pich A., Haverich A., Wilhelmi M. (2011). The effect of detergent-based decellularization procedures on cellular proteins and immunogenicity in equine carotid artery grafts. *Biomaterials*.

[70] Zhang J., Hu Z. Q., Turner N. J. (2016). Perfusion-decellularized skeletal muscle as a three-dimensional scaffold with a vascular network template. *Biomaterials*.

[71] Giraldo-Gomez D. M., Leon-Mancilla B., Del Prado-Audelo M. L. (2016). Trypsin as enhancement in cyclical tracheal decellularization: morphological and biophysical characterization. *Materials Science and Engineering C*.

[72] Mendes R. L., Nobre B. P., Cardoso M. T., Pereira A. P., Palavra A. F. (2003). Supercritical carbon dioxide extraction of compounds with pharmaceutical importance from microalgae. *Inorganica Chimica Acta*.

[73] Kim S. A., Kim O. Y., Rhee M. S. (2010). Direct application of supercritical carbon dioxide for the reduction of Cronobacter spp. (Enterobacter sakazakii) in end products of dehydrated powdered infant formula. *Journal of Dairy Science*.

[74] Jung W. Y., Choi Y. M., Rhee M. S. (2009). Potential use of supercritical carbon dioxide to decontaminate Escherichia coli O157:H7, Listeria monocytogenes, and Salmonella typhimurium in alfalfa sprouted seeds. *International Journal of Food Microbiology*.

[75] Brown B. N., Freund J. M., Han L. (2011). Comparison of three methods for the derivation of a biologic scaffold composed of adipose tissue extracellular matrix. *Tissue Engineering—Part C: Methods*.

[76] Flynn L. E. (2010). The use of decellularized adipose tissue to provide an inductive microenvironment for the adipogenic differentiation of human adipose-derived stem cells. *Biomaterials*.

[77] Luo L., Eswaramoorthy R., Mulhall K. J., Kelly D. J. (2015). Decellularization of porcine articular cartilage explants and their subsequent repopulation with human chondroprogenitor cells. *Journal of the Mechanical Behavior of Biomedical Materials*.

[78] Cortiella J., Niles J., Cantu A. (2010). Influence of acellular natural lung matrix on murine embryonic stem cell differentiation and tissue formation. *Tissue Engineering Part A*.

[79] Wong M. L., Griffiths L. G. (2014). Immunogenicity in xenogeneic scaffold generation: antigen removal vs. decellularization. *Acta Biomaterialia*.

[80] Boeer U., Buettner F. F. R., Klingenberg M. (2014). Immunogenicity of intensively decellularized equine carotid arteries is conferred by the extracellular matrix protein collagen type VI. *PLoS ONE*.

[81] Wang F., Guan J. (2010). Cellular cardiomyoplasty and cardiac tissue engineering for myocardial therapy. *Advanced Drug Delivery Reviews*.

[82] Gong Y. Y., Xue J. X., Zhang W. J., Zhou G. D., Liu W., Cao Y. (2011). A sandwich model for engineering cartilage with acellular cartilage sheets and chondrocytes. *Biomaterials*.

[83] Xue J. X., Gong Y. Y., Zhou G. D., Liu W., Cao Y., Zhang W. J. (2012). Chondrogenic differentiation of bone marrow-derived mesenchymal stem cells induced by acellular cartilage sheets. *Biomaterials*.

[84] Chen H. J., Wei Z., Sun J. (2016). A recellularized human colon model identifies cancer driver genes. *Nature Biotechnology*.

[85] Ingram J. H., Korossis S., Howling G., Fisher J., Ingham E. (2007). The use of ultrasonication to aid recellularization of acellular natural tissue scaffolds for use in anterior cruciate ligament reconstruction. *Tissue Engineering*.

[86] Crabbé A., Liu Y., Sarker S. F. (2015). Recellularization of decellularized lung scaffolds is enhanced by dynamic suspension culture. *PLoS ONE*.

[87] Farag A., Vaquette C., Theodoropoulos C., Hamlet S. M., Hutmacher D. W., Ivanovski S. (2014). Decellularized periodontal ligament cell sheets with recellularization potential. *Journal of Dental Research*.

[88] Bourget J.-M., Gauvin R., Larouche D. (2012). Human fibroblast-derived ECM as a scaffold for vascular tissue engineering. *Biomaterials*.

[89] Dunne L. W., Huang Z., Meng W. (2014). Human decellularized adipose tissue scaffold as a model for breast cancer cell growth and drug treatments. *Biomaterials*.

[90] Wang L., Johnson J. A., Zhang Q., Beahm E. K. (2013). Combining decellularized human adipose tissue extracellular matrix and adipose-derived stem cells for adipose tissue engineering. *Acta Biomaterialia*.

[91] Datta N., Holtorf H. L., Sikavitsas V. I., Jansen J. A., Mikos A. G. (2005). Effect of bone extracellular matrix synthesized in vitro on the osteoblastic differentiation of marrow stromal cells. *Biomaterials*.

[92] Kang H., Peng J., Lu S. (2014). In vivo cartilage repair using adipose-derived stem cell-loaded decellularized cartilage ECM scaffolds. *Journal of Tissue Engineering and Regenerative Medicine*.

[93] Goh S.-K., Bertera S., Olsen P. (2013). Perfusion-decellularized pancreas as a natural 3D scaffold for pancreatic tissue and whole organ engineering. *Biomaterials*.

[94] GonfiOtti A., Jaus M. O., Barale D. (2014). The first tissue-engineered airway transplantation: 5-year follow-up results. *The Lancet*.

